# DoF-Dependent and Equal-Partition Based Lens Distortion Modeling and Calibration Method for Close-Range Photogrammetry

**DOI:** 10.3390/s20205934

**Published:** 2020-10-20

**Authors:** Xiao Li, Wei Li, Xin’an Yuan, Xiaokang Yin, Xin Ma

**Affiliations:** 1 School of Mechanical and Electrical Engineering, China University of Petroleum (East China), Huangdao, Qingdao 266580, China; lix2020@upc.edu.cn (X.L.); xinancom@upc.edu.cn (X.Y.); xiaokang.yin@upc.edu.cn (X.Y.); 2 Polytechnic Institute, Purdue University, West Lafayette, IN 47907, USA; ma633@purdue.edu

**Keywords:** lens distortion, DoF-dependent, distortion partition, vision measurement

## Abstract

Lens distortion is closely related to the spatial position of depth of field (DoF), especially in close-range photography. The accurate characterization and precise calibration of DoF-dependent distortion are very important to improve the accuracy of close-range vision measurements. In this paper, to meet the need of short-distance and small-focal-length photography, a DoF-dependent and equal-partition based lens distortion modeling and calibration method is proposed. Firstly, considering the direction along the optical axis, a DoF-dependent yet focusing-state-independent distortion model is proposed. By this method, manual adjustment of the focus and zoom rings is avoided, thus eliminating human errors. Secondly, considering the direction perpendicular to the optical axis, to solve the problem of insufficient distortion representations caused by using only one set of coefficients, a 2D-to-3D equal-increment partitioning method for lens distortion is proposed. Accurate characterization of DoF-dependent distortion is thus realized by fusing the distortion partitioning method and the DoF distortion model. Lastly, a calibration control field is designed. After extracting line segments within a partition, the de-coupling calibration of distortion parameters and other camera model parameters is realized. Experiment results shows that the maximum/average projection and angular reconstruction errors of equal-increment partition based DoF distortion model are 0.11 pixels/0.05 pixels and 0.013°/0.011°, respectively. This demonstrates the validity of the lens distortion model and calibration method proposed in this paper.

## 1. Introduction

Vision measurement is a subject that allows quantitative perception of scene information by combining image processing with calibrated camera parameters. Therefore, the calibration accuracy of the parameters is an important determinant of the vision measurement uncertainty. The lens distortion is closely related to the depth of field (DoF), which refers to the distance between the nearest and the farthest objects that are in acceptably sharp focus in an image. For medium- or high-accuracy applications, close-range imaging parameters (e.g., short object distance (<1 m) and small focal length) are often adopted. In such occasions, DoF has a significant influence on lens distortion and, hence, becomes a major cause of vision measurement errors. For instance, to ensure that the vision has a micron-level accuracy when detecting the contouring error of a machine tool [1], the camera is placed 400 mm away from the focal plane to collect and analyze the image sequence of the interpolation trajectory running in the DoF. In this case, measurement errors ranging from dozens to hundreds of microns can be caused by a large lens distortion. Therefore, to improve the vision measurement accuracy in close-range photogrammetry, accurate modeling and calibration of the DoF-dependent lens distortion are urgently needed.

The lens distortion model maps the relation between distorted and undistorted image points. Models to show the relations vary according to the types of optical systems, which include the polynomial distortion model, logarithmic fish-eye distortion model [2], polynomial fish-eye distortion model [2,3,4,5], field-of-view (FoV) distortion model [6], division distortion model [7,8], rational function distortion model [9,10], and so on. In 1971, Brown [11,12] proposed the Gaussian polynomial function to express radial and decentering distortion, which is particularly suitable for studying the distortion of a standard lens in high-accuracy measurements [13,14]. Later, researchers noticed that the observed radial and decentering distortion varies with the focal length, the lens focusing state (i.e., focused or defocused), and the DoF position. Since then, researchers have focused on the improvement of distortion calibration and modeling methods to obtain a precise representation of distortion behavior. For the distortion calibration, the study goes in two directions: the coupled-calibration method and the decoupled-calibration method. The former can be generally divided into three types: self-calibration method [15], active calibration method, and traditional calibration method [16]. Among the traditional ones, Zhang’s calibration method [17] and its improved method [18,19,20], used widely in industry and scientific research, are the most popular. In this coupled-calibration method, the distortion parameters are calculated by performing a full-scale optimization for all parameters. Due to the strong coupling effect, the estimated errors of other parameters (i.e., intrinsic and extrinsic parameters) in the camera model would be propagated to that of distortion parameters, thus leading to the failure of getting optimal solutions. By contrast, the decoupled-calibration method does not involve coupling other factors or entail any prior geometric knowledge of the calibration object, and only geometric invariants of some image features, such as straight lines [6,12,21,22,23], vanishing points [24], or spheres [25], are needed to solve the parameters. Among these features, straight lines can be easily reflected in scenes and extracted from noise images, thus having enormous potential.

Regarding the distortion modeling, some researchers incorporated the DoF into the distortion function. Magill [26] used the distortion of two focal planes at infinity to solve that of an arbitrary focal plane. Then, Brown [12] improved Magill’s model by establishing distortion models of any focal plane and any defocused plane (the plane perpendicular to the optical axis in the DoF) on the condition that the distortions of two focal planes are known. Soon after, Fryer [27], based on Brown’s model, realized the lens distortion calibration of an underwater camera [28]. Fraser and Shortis [29] introduced an empirical model and solved the Brown model’s problem of inaccurate description of large image distortion. Additionally, Dold [30] established a DoF distortion model that is different from Brown’s and solved the model parameters through the strategy of bundle adjustment. In 2004, Brakhage [31] characterized the DoF distortion of the telecentric lens in a fringe projection system by using Zernike Polynomials. Moreover, in 2006, the DoF distortion distribution of the grating projection system was experimentally analyzed by Bräuer-Burchardt. In 2008, Hanning [32] introduced depth (object distance) into the spline function to form a distortion model and used the model to calibrate radial distortion. 

The above DoF distortion models not only depend on the focusing state but also relate to the distortion coefficients on the focal plane. For these models, on the one hand, the focusing state is usually adjusted by manually twisting the zoom and focus rings, which introduces human errors and changes the camera parameters. On the other hand, the focus distance and distortion parameters on the focal plane cannot be determined accurately. To overcome the problem, Alvarez [33], based on Brown’s and Fraser’s models, deduced a radial distortion model that is suitable for planar scenarios. With this model, when the focal length is locked, distortion at any image position can be estimated by using two lines in a single photograph. In 2017, Dong [34] proposed a DoF distortion model, by which the researcher accurately calibrated the distortion parameters on arbitrary object planes, and reduced the error from 0.055 mm to 0.028 mm in the measuring volume of 7.0 m × 3.5 m × 2.5 m with the large-object-distance of 6 m. Additionally, in 2019, Ricolfe-Viala [35] proposed a depth-dependent high distortion lens calibration method, by embedding the object distance in the division distortion model, and the highly distorted images can be corrected with only one distortion parameter. However, these researchers only used one set of coefficients, which is not sufficient to accurately represent the distortion. To address this problem, some scholars adopted the idea of partitioning to process image distortion, which uses several sets of distortion coefficients to characterize the distortion. The study, however, which is only applicable to the partitioning of a 2D object plane, fails to take into account the distortion partition within the DoF and the correlation between lens distortion and DoF. Our previous work partitioned the distortion with an equal radius [36]. Although it improved the vision measurement accuracy, the distortion correction accuracy within the partition corresponding to the image edge is still low. Besides, the distortion model we adopted depends on the focusing state of the lens, thus is less practical. In general, the current distortion model and partitioning method cannot accurately reflect the lens DoF distortion behavior in close-range photography, especially for short-distance measurements.

To solve the above problems, the lens distortion model and calibration method for short-distance measurement, which takes into consideration the dimensions of DoF and equal-increment partition of distortion, are proposed in this paper. The rest of this paper is organized as follows. In Section 2, a focusing-state-independent DoF distortion model, which only involves the spatial position of the observed point, is constructed. In Section 3, based on the model in previous section, an equal-increment partitioning DoF distortion model is proposed, which enables a fine representation of the lens distortion in the photographic field. Section 4 details the calibration method for both DoF distortion and camera model parameters, as well as the image processing of the control field for distortion calibration. In Section 5, experimental verification of the proposed lens distortion model and calibration method is carried out. Finally, Section 6 concludes this paper.

## 2. Focusing-State-Independent DoF Distortion Model

The observed distortion of a point varies with its position within the DoF. Though the close-range imaging configuration increases the visible range, it enlarges the DoF image distortion, consequently affecting the measurement accuracy. To break the limitations of the aforementioned in-plane and DoF distortion model in the vision measurement of short-distance and small-focal-length settings, a DoF-dependent yet focusing-state-independent distortion model is proposed in this paper.

### 2.1. Pinhole Camera Model with Distortion

**Figure 1:**
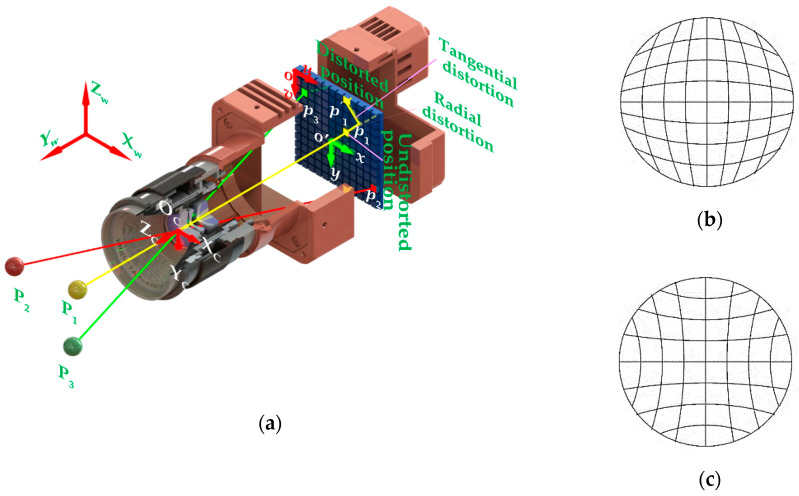
Schematic diagram of camera model and lens distortion: (**a**) camera model; (**b**) barrel distortion; (**c**) pincushion distortion.

As illustrated in Figure 1, the linear pinhole camera model depicts the one-to-one mapping between the 3D points in the object space and its 2D projections in the image. Let $p(\begin{array}{cc} u_{li} & v_{li} \end{array})$ be undistorted coordinates mapped from a spatial point in the world coordinate system $O_{w}X_{w}Y_{w}Z_{w}$ to the image coordinate system ouv through the optical center $O_{C}$. Then, camera mapping can be expressed as [17]

(1) $$z^{\prime }\left[\begin{array}{c} u_{li}\\ v_{li}\\ 1 \end{array}\right]=K\cdot M\cdot \left[\begin{array}{c} X_{w}\\ Y_{w}\\ Z_{w}\\ 1 \end{array}\right]$$

where $z^{\prime }$ describes the scaling factor; K is the intrinsic parameter matrix, which quantitatively characterizes the critical parameters of the image sensor (i.e., Charge Coupled Device (CCD) or Complementary Metal-Oxide Semiconductor (CMOS)); Matrix M, expressing the transformation between the vision coordinate system (VCS) and the world coordinate system, consists of the rotation matrix R and translation matrix T.

However, manufacturing and assembly errors can lead to radial and decentering lens distortion. Consequently, the pinhole assumption does not hold for real camera systems, and the image projection of a straight line would be bent into a curve (Figure 1b,c). To characterize the lens distortion, Brown proposed the distortion model in a polynomial form [11,12]:

(2) $$\{\begin{array}{c} u_{li}={\bar{u}}_{li}+\delta_{u_{li}}\\ v_{li}={\bar{v}}_{li}+\delta_{v_{li}}\\ \delta_{u_{li}}={\bar{u}}_{li}\cdot (1+K_{1}\cdot r^{2}+K_{2}\cdot r^{4}+\cdots )+\left[P_{1}\cdot (r^{2}+2\cdot {\bar{u}}_{li}{}^{2})+2P_{2}\cdot {\bar{u}}_{li}\cdot {\bar{v}}_{li}\right]\cdots \\ \delta_{v_{li}}={\bar{v}}_{li}\cdot (1+K_{1}\cdot r^{2}+K_{2}\cdot r^{4}+\cdots )+\left[P_{2}\cdot (r^{2}+2\cdot {\bar{v}}_{li}{}^{2})+2P_{1}\cdot {\bar{u}}_{li}\cdot {\bar{v}}_{li}\right]\cdots \end{array}$$

where $\left(\begin{array}{cc} {\bar{u}}_{li} & {\bar{v}}_{li} \end{array}\right)$ is the distorted coordinates; $\delta_{u_{li}}$ and $\delta_{v_{li}}$ are the distortion functions of an image point in the u and v direction respectively; $\left(\begin{array}{cc} u_{0} & v_{0} \end{array}\right)$ denotes the distortion center; $r=\sqrt{{\left({\bar{u}}_{li}-u_{0}\right)}^{2}+{\left({\bar{v}}_{li}-v_{0}\right)}^{2}}$ stands for the distortion radius of the image point; $K_{1}$ and $K_{2}$ are the first and second-order coefficients of radial distortion respectively; while, $P_{1}$ and $P_{2}$ are the first and second-order coefficients of decentering distortion respectively.

### 2.2. Distortion Model in the Focal Plane

#### 2.2.1. Radial Distortion Model

Let $\delta r_{\infty }$ be the radial distortion for a lens that is focused on plus infinity, while $\delta r_{-\infty }$ on the minus infinity. $m_{s}$ refers to the vertical magnification in the focal plane at object distance s. According to Magill’s model [26], $\delta r_{s}$, the lens radial distortion in the focal plane, can be expressed as

(3) $$\delta r_{s}=\delta r_{-\infty }-m_{s}\cdot \delta r_{\infty }$$

Let $\delta r_{s_{m}}$ and $\delta r_{s_{k}}$ be the radial distortions in the focal planes when the lens is focused on the distances of $s_{m}$ and $s_{k}$ respectively. Then, the distortion function for focal plane $\delta r_{s}$ at distance s can be written as

(4) $$\delta r_{s}=\alpha_{s}\cdot \delta r_{s_{m}}+\left(1-\alpha_{s}\right)\cdot \delta r_{s_{k}}$$

where, f is the focal length; $\alpha_{s}=\frac{\left(s_{k}-s_{m})\cdot (s-f\right)}{\left(s_{k}-s)\cdot (s_{m}-f\right)}$. The i-th radial distortion coefficients $K_{i}^{s}$ for focused object plane at distance s are

(5) $$K_{i}^{s}=\alpha_{s}\cdot K_{i}^{s_{m}}+\left(1-\alpha_{s}\right)\cdot K_{i}^{s_{k}}\;i=1,2.$$

where $K_{i}^{s_{m}}$ and $K_{i}^{s_{k}}$ are the i-th radial distortion coefficients when the lens is focused on the distances of $s_{m}$ and $s_{k}$ respectively. As can be easily noticed in Equation (5), if the radial distortion coefficients of two different focal planes are known, the radial distortion coefficients of any focal plane can be obtained.

#### 2.2.2. Decentering Distortion

As for the decentering distortion, the equations are as follows [12]:

(6) {δru=(1−fs)(P1⋅(r2+2u2)+2P2⋅u⋅v)δrv=(1−fs)(P1⋅(r2+2v2)+2P2⋅u⋅v)rs,s′=s−fs′−f⋅s′s

where (1−fs)⋅rs,s′ is the compensation coefficient; $\delta r_{u}$ and $\delta r_{v}$ represent the components of the decentering distortion in the u and the v direction respectively; s and $s^{\prime }$ depict the object distances corresponding to the two focal planes, respectively.

### 2.3. DoF-Dependent Distortion Model for Arbitrary Defocused Plane

#### 2.3.1. DoF-Dependent Radial Distortion Model

Fraser and Shortis [29] proposed an empirical model for describing the distortion of any object plane (or defocused plane), which solved Brown model’s problem of inaccurate description of severe distortion caused by the image configuration of short-distance and small-focal-length settings. The equation is as follows:

(7) $$K^{s,s_{p}}=K^{s}+g\cdot (K^{s_{p}}-K^{s})$$

where $K^{s,s_{p}}$ denotes the radial distortion coefficient in the defocused plane with the depth of $s_{p}$ when the lens is focused at distance s; g is the empirical coefficient; $K^{s_{p}}$ and $K^{s}$ represent the radial distortion coefficients in the focal planes at distances $s_{p}$ and s respectively. By extending the equation, we can get the radial distortion function $\delta r^{s,s_{n}}$ at $s_{n}$ expressed by the $\delta r^{s,s_{m}}$ at $s_{m}$ when the lens is focused at the distance of s:

(8) $$\{\begin{array}{c} \delta r^{s,s_{m}}=\delta r^{s}+g\cdot (\delta r^{s_{m}}-\delta r^{s})\\ \delta r^{s,s_{n}}-\delta r^{s,s_{m}}=\delta r^{s}+g\cdot (\delta r^{s_{n}}-\delta r^{s})-\delta r^{s}-g\cdot (\delta r^{s_{m}}-\delta r^{s}) \end{array}$$

From the above equation, we can easily obtain $\delta r^{s,s_{n}}=\delta r^{s,s_{m}}+\alpha_{s,s_{m}(s_{n})}\cdot (\delta r^{s}-\delta r^{s,s_{m}})$. Then, by extending the results to the radial distortion of a point in the defocused plane at distance $s_{k}$, the relationship between $\delta r^{s,s_{n}}$, $\delta r^{s,s_{m}}$ and $\delta r^{s,s_{k}}$ can be given by

(9) $$\left\{ \begin{array}{l} \delta r^{s,s_{n}}=\delta r^{s,s_{m}}+\alpha_{s,s_{m}(s_{n})}\cdot (\delta r^{s}-\delta r^{s,s_{m}})\\ \delta r^{s,s_{n}}=\delta r^{s,s_{k}}+\alpha_{s,s_{k}(s_{n})}\cdot (\delta r^{s}-\delta r^{s,s_{k}})\\ \delta r^{s,s_{m}}=\delta r^{s,s_{k}}+\alpha_{s,s_{k}(s_{m})}\cdot (\delta r^{s}-\delta r^{s,s_{k}}) \end{array}\right.$$

in which $\alpha_{s,s_{m}(s_{n})}=\frac{s_{m}-s_{n}}{s_{m}-s}\cdot \frac{s-f}{s_{n}-f}$, $\alpha_{s,s_{k}(s_{n})}=\frac{s_{k}-s_{n}}{s_{k}-s}\cdot \frac{s-f}{s_{n}-f}$, and $\alpha_{s,s_{k}(s_{m})}=\frac{s_{k}-s_{m}}{s_{k}-s}\cdot \frac{s-f}{s_{m}-f}$.

After eliminating the focus distance and the distortion in the focal plane, we can obtain the following equation:

(10) $$\left\{ \begin{array}{l} \delta r^{s,s_{m}}=\delta r^{s,s_{k}}+\frac{s_{k}-s_{m}}{s_{k}-s}\cdot \frac{s-f}{s_{m}-f}\cdot (\delta r^{s}-\delta r^{s,s_{k}})\\ \delta r^{s,s_{n}}=\delta r^{s,s_{k}}+\frac{s_{k}-s_{n}}{s_{k}-s}\cdot \frac{s-f}{s_{n}-f}\cdot (\delta r^{s}-\delta r^{s,s_{k}}) \end{array}\right.$$

Then, we can have $\frac{s-f}{s_{k}-s}\cdot (\delta r^{s}-\delta r^{s,s_{k}})=(\delta r^{s,s_{m}}-\delta r^{s,s_{k}})\cdot \frac{s_{m}-f}{s_{k}-s_{m}}$. $\delta r^{s,s_{n}}$ can be expressed as

(11) $$\delta r^{s,s_{n}}=\frac{\delta r^{s,s_{k}}\cdot (s_{m}-f)\cdot (s_{k}-s_{m})+(s_{k}-s_{n})\cdot (s_{k}-f)\cdot (\delta r^{s,s_{m}}-\delta r^{s,s_{k}})}{\left(s_{m}-f)\cdot (s_{k}-s_{m}\right)}$$

Obviously, when the lens is focused at distance s, through two distortions corresponding to object distances $s_{m}$ and $s_{k}$ respectively, radial distortion coefficient in any defocused plane with the depth of $s_{n}$ when the lens is focused at distance s can be obtained:

(12) $$K_{i}^{s,s_{n}}=\frac{K_{i}^{s,s_{k}}\cdot (s_{m}-f)\cdot (s_{k}-s_{m})+(s_{k}-s_{n})\cdot (s_{k}-f)\cdot (K_{i}^{s,s_{m}}-K_{i}^{s,s_{k}})}{\left(s_{m}-f)\cdot (s_{k}-s_{m}\right)}i=1\cdots 2.$$

When two object planes are set, $K_{i}^{s,s_{m}}$, $K_{i}^{s,s_{k}}$, $s_{m}$, $s_{k}$ and f are known. Thus, $K_{i}^{s,s_{n}}$ in Equation (12) is only dependent on $s_{n}$, and it is independent of the distortion coefficient $K_{i}^{s}$ on the focal plane and the focus distance s.

#### 2.3.2. DoF-Dependent Decentering Distortion Model

In Equation (6), since $\delta P^{s,s_{m}}=r^{s,s_{m}}\cdot \delta P^{\infty }$, the distortion in the focal plane can be written as

(13) $$\{\begin{array}{c} \delta P^{s,s_{m}}=(1-\frac{f}{s})\cdot \frac{s-f}{s_{m}-f}\cdot \frac{s_{m}}{s}\cdot \delta P^{\infty }\\ \delta P^{s,s_{k}}=(1-\frac{f}{s})\cdot \frac{s-f}{s_{k}-f}\cdot \frac{s_{k}}{s}\cdot \delta P^{\infty }\\ \delta P^{s,s_{n}}=(1-\frac{f}{s})\cdot \frac{s-f}{s_{n}-f}\cdot \frac{s_{n}}{s}\cdot \delta P^{\infty } \end{array}$$

where $\delta P^{s,s_{m}}$, $\delta P^{s,s_{k}}$ and $\delta P^{s,s_{n}}$ are the decentering distortion functions in the defocused plane at the object distances of $s_{m}$, $s_{k}$ and $s_{n}$ when the lens is focused at distance s respectively. From the first two lines of the above equation, we get $\frac{\delta P^{s,s_{k}}}{\delta P^{s,s_{m}}}=\frac{s-f}{s_{k}-f}\cdot \frac{s_{m}-f}{s-f}\cdot \frac{s_{k}}{s}\cdot \frac{s}{s_{m}}=\frac{s_{m}-f}{s_{k}-f}\cdot \frac{s_{k}}{s_{m}}=M^{s_{k},s_{m}}$, and then

(14) $$\{\begin{array}{c} f=\frac{1-M^{s_{k},s_{m}}}{s_{k}-s_{m}\cdot M^{s_{k},s_{m}}}\\ \frac{\delta P^{s,s_{n}}}{\delta P^{s,s_{m}}}=\frac{s_{m}-f}{s_{n}-f}\cdot \frac{s_{n}}{s_{m}} \end{array}$$

Put the first line into the second one, and we obtain

(15) $$\frac{\delta P^{s,s_{n}}}{\delta P^{s,s_{m}}}=\frac{s_{m}-\frac{1-M^{s_{k},s_{m}}}{s_{k}-s_{m}\cdot M^{s_{k},s_{m}}}}{s_{n}-\frac{1-M^{s_{k},s_{m}}}{s_{k}-s_{m}\cdot M^{s_{k},s_{m}}}}\cdot \frac{s_{n}}{s_{m}}=\frac{s_{m}\cdot s_{k}-s_{m}^{2}\cdot M^{s_{k},s_{m}}-1+M^{s_{k},s_{m}}}{s_{n}\cdot s_{k}-s_{m}^{}\cdot s_{n}^{}\cdot M^{s_{k},s_{m}}-1+M^{s_{k},s_{m}}}\cdot \frac{s_{n}}{s_{m}}$$

Equation (15) can be simplified to

(16) $$\{\begin{array}{c} \delta P^{s,s_{n}}=\frac{M^{s_{k},s_{m}}\cdot (1-s_{m}^{2})+(s_{m}\cdot s_{k}-1)}{M^{s_{k},s_{m}}\cdot (1-s_{n}\cdot s_{m})+s_{n}\cdot s_{k}-1}\cdot \frac{s_{n}}{s_{m}}\cdot \delta P^{s,s_{m}}\\ P_{i}^{s,s_{n}}=\frac{M^{s_{k},s_{m}}\cdot (1-s_{m}^{2})+(s_{m}\cdot s_{k}-1)}{M^{s_{k},s_{m}}\cdot (1-s_{n}\cdot s_{m})+s_{n}\cdot s_{k}-1}\cdot \frac{s_{n}}{s_{m}}\cdot P_{i}^{s,s_{m}}\;i=1,2. \end{array}$$

Put Msk,sm=Pis,skPis,sm (i=1,2) into Equation (16), and the following equation is obtained:

(17) $$P_{i}^{s,s_{n}}=\frac{P_{i}^{s,s_{k}}\cdot (1-s_{m}^{2})+P_{i}^{s,s_{m}}\cdot (s_{m}\cdot s_{k}-1)}{P_{i}^{s,s_{k}}\cdot (1-s_{m}\cdot s_{n})+P_{i}^{s,s_{m}}(s_{n}\cdot s_{k}-1)}\cdot \frac{s_{n}}{s_{m}}\cdot P_{i}^{s,s_{m}}i=1,2.$$

Given the parameters $P_{i}^{s,s_{m}}$, $P_{i}^{s,s_{k}}$, $s_{m}$ and $s_{k}$ are known, it can be illustrated from Equation (17) that the decentering distortion coefficient $P_{i}^{s,s_{n}}$ in any defocused plane is dependent only on the object distance, $s_{n}$, and is independent of the focus distance s and the distortion $P_{i}^{s}$ in the focal plane. Moreover, since focal length f is not included in Equation (17), decentering distortion is not affected by this parameter.

Hereto, the DoF-dependent yet focusing-state-independent distortion model suitable for close-range, short-distance measurement scenes is established, which overcomes the limited practicability caused by the way of calibrating DoF distortion by manual adjustment of the focus and zoom rings, and it also solves the problem when the current position and the distortion parameters of the focal plane are not exactly known.

## 3. Equal-Increment Partition Based DoF Distortion Model

The distortion coefficients are solved by minimizing the straightness error of the observed points. If a set of distortion coefficients is used to describe the distortion in the whole image, the distortion coefficients will be the error balance of all points. However, for each region of the image the error is not the minimum. Hence, an equal-increment partition based DoF distortion model is proposed in this section. The distortion spreads outward from the image center along a circumferential contour, with the characteristics of the image being small in the middle and large on the image edge. In this paper, we first partition the in-plane distortion in an equal-increment way, then the 2D partition strategy is extended to the 3D photographic field.

### 3.1. Equal-Increment Based Distortion Partitioning Method

**Figure 2:**
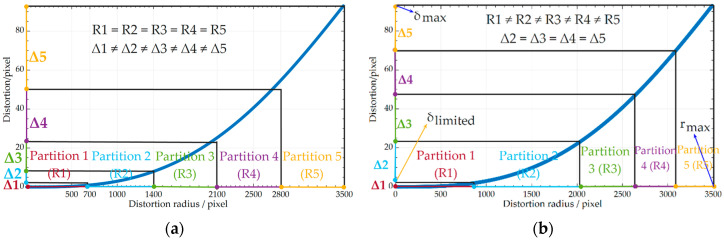
Two distortion partitioning methods: (**a**) equal-radius partition; (**b**) equal-increment partition.

Figure 2 presents two distortion partitioning methods. The X axis represents the distance from an image point to the distortion center $\left(u_{0},v_{0}\right)$, namely the distortion radius. The Y axis describes the distortion in pixels. The blue curve is the distortion curve calculated by the features in the whole image. As illustrated in Figure 2a, when DoF distortion is partitioned by an equal radius, the distortion increment of each partition is different ($\Delta_{1}<\Delta_{2}<\Delta_{3}<\Delta_{4}<\Delta_{5}$) despite the same distortion radius increment ($R_{1}=R_{2}=R_{3}=R_{4}=R_{5}$) [36]. For a polynomial-based distortion function, it is well known that the more scattered the distorted points and the larger the distortion increments are, the lower the regression accuracy of the function to the distortion is. As a result, the estimated accuracy of the partition’s distortion parameters decreases gradually from inside to outside ($\varepsilon_{1}>\varepsilon_{2}>\varepsilon_{3}>\varepsilon_{4}>\varepsilon_{5}$).

To solve the problem, a DoF distortion model based on the equal-increment partition is proposed in this paper, and the procedures are as follows:

(1) Estimate the distortion curve using all features in the whole image (Figure 2b). Then, determine the maximum value of image distortion $\delta_{max}$ according to the maximum distortion radius and distortion curves. The maximum distortion radius of the image is $r_{\max}=\frac{1}{2}\cdot \sqrt{{\left(I_{l}-u_{0}\right)}^{2}+{\left(I_{h}-v_{0}\right)}^{2}}$, where $I_{l}$ and $I_{h}$ are the length and height of the image, respectively.
(2) In the central image region, the distortion is so tiny that it cannot converge after iteration, which results in a poorer quality of the undistorted image than that of the original one. Therefore, we use $\delta_{limited}$, the minimum distortion value when the algorithm converges in the central image region, as the threshold to estimate $r_{limited}$, the minimum value of the image distortion radius.
(3) Determine the number of partitions $n_{p}$.
(4) Use the maximum distortion $\delta_{max}$, the lower-limit distortion $\delta_{limited}$, and $n_{p}$ to determine the distortion increment of each partition $\delta_{equ}=\left(\delta_{\max}-\delta_{limited}\right)/\left(n_{p}-1\right)$, $\delta_{equ}=\Delta_{2}=\Delta_{3}=\Delta_{4}=\Delta_{5}$ (Figure 2b).
(5) Calculate the radius increment of each partition using $\delta_{equ}$ and the distortion curve, $R_{1}\neq R_{2}\neq R_{3}\neq R_{4}\neq R_{5}$ (Figure 2b).
(6) Calibrate the distortion curve of each partition by the features in the corresponding partition of the image utilizing the decoupled-calibration method (see Section 4).

Then the distortion partition of the 2D object plane is extended to the 3D DoF. As can be known from Equation (1), the object-to-image mapping satisfies the following:

(18) $$\{\begin{array}{c} x=f\cdot \frac{X_{m}}{Z_{m}}=f\cdot \frac{X_{k}}{Z_{k}}\\ y=f\cdot \frac{Y_{m}}{Z_{m}}=f\cdot \frac{Y_{k}}{Z_{k}} \end{array}$$

where $P_{m}(\begin{array}{ccc} X_{m} & Y_{m} & Z_{m} \end{array})$ and $P_{k}(\begin{array}{ccc} X_{k} & Y_{k} & Z_{k} \end{array})$ are two points in the VCS. The 2D point $p(\begin{array}{cc} x & y \end{array})$ (in millimeters) is the image projection of the $P_{m}$ and $P_{k}$ ($P_{m}$, $P_{k}$, and $O_{C}$ are collinear). Let ρ be the partition radius, then $x^{2}+y^{2}=\rho^{2}$, and we get

(19) $$\{\begin{array}{c} f^{2}\cdot \frac{X_{m}^{2}}{Z_{m}^{2}}+f^{2}\cdot \frac{Y_{m}^{2}}{Z_{m}^{2}}=\rho^{2}\\ f^{2}\cdot \frac{X_{k}^{2}}{Z_{k}^{2}}+f^{2}\cdot \frac{Y_{k}^{2}}{Z_{k}^{2}}=\rho^{2} \end{array}$$

**Figure 3:**
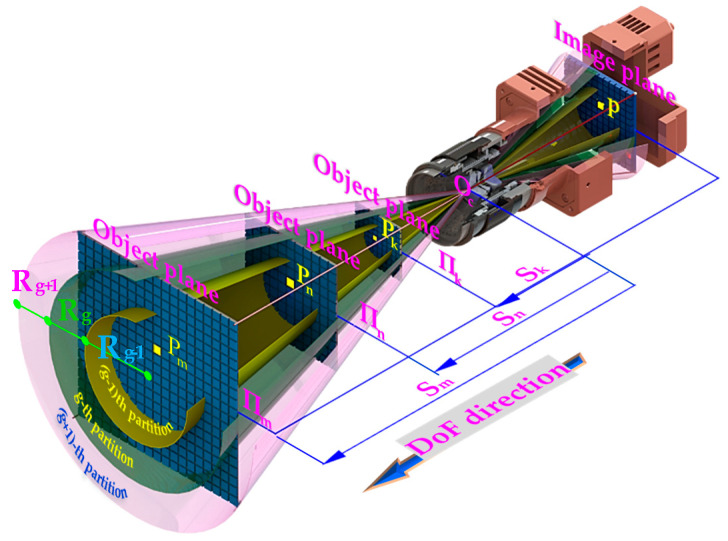
The geometric relationship between the partition radii in different object planes.

From the above equation, we can know that $f\cdot R_{m}=\rho \cdot Z_{m}$ and $Z_{m}\cdot R_{k}=Z_{k}\cdot R_{m}$, where $Z_{m}$ and $Z_{k}$ are the depths of the m-th ($\Pi_{m}$) and the k-th ($\Pi_{k}$) object planes in the VCS respectively. $R_{m}$ and $R_{k}$ are the partition radius of the two object planes. Let $s_{m}=Z_{m}$ and $s_{k}=Z_{k}$, and then extend the above distortion partitions to 3D DoF domain. As shown in Figure 3, if the range of the g-th partition in the object plane $\Pi_{m}$ is $\left[\begin{array}{cc} (g-1)\cdot R_{m} & g\cdot R_{m} \end{array}\right]$, the partition range in object planes $\Pi_{k}$ and $\Pi_{n}$ are $\left[\begin{array}{cc} \left(g-1)\cdot (s_{k}\cdot R_{m}/s_{m}\right) & g\cdot (s_{k}\cdot R_{m}/s_{m}) \end{array}\right]$ and $\left[\begin{array}{cc} \left(g-1)\cdot (s_{n}\cdot R_{m}/s_{m}\right) & g\cdot (s_{n}\cdot R_{m}/s_{m}) \end{array}\right]$ respectively. In this way, although the distortion radius in each partition is different, distortion coefficients can be obtained with high accuracy when the image distortion is partitioned by equal distortion increments.

### 3.2. Equal-Increment Partition Based DoF Distortion Model

After partitioning the DoF distortion, we incorporate the partitions into the DoF distortion model. Procedures to solve the partition radius and distortion coefficients on any object distance $s_{n}$ are as follows:

(1) Partition the distortion in the object plane $\Pi_{m}$ using the proposed method, and calculate the i-th order radial and decentering distortion coefficients in the g-th partition. Register the two coefficients as Kis,smg and Pis,smg respectively.
(2) Based on the g-th partition in the object plane $\Pi_{m}$ (the object distance is $s_{m}$), the corresponding partition radius in the object plane $\Pi_{k}$ (the object distance is $s_{k}$) is calculated. In addition, the i-th order radial and decentering distortion coefficients can be computed. Register the two coefficients as Kis,skg and Pis,skg respectively.
(3) Based on the partitions in the object plane $\Pi_{m}$, we calculate the partitions in the object distance plane $\Pi_{n}$ (the object distance is $s_{n}$). Then, for the g-th partition of the object plane $\Pi_{n}$, the radial distortion coefficient Kis,sng and the decentering distortion coefficient Pis,sng can be expressed as
  (20) {Kis,sng=f(Kis,smg,Kis,skg,sn)Pis,sng=f(Pis,smg,Pis,skg,sn)
  From the equation, we can know
  (21) {Kis,sng=Kis,skg⋅(sm−f)⋅(sk−sm)+(sk−sn)⋅(sk−f)⋅(Kis,smg−Kis,skg)(sm−f)⋅(sk−sm)  Pis,sng=Pis,skg⋅(1−sm2)+Pis,smg⋅(sm⋅sk−1)Pis,skg⋅(1−sm⋅sn)+Pis,smg(sn⋅sk−1)⋅snsm⋅Pis,smg  i=1,2  g=1,2,⋯,np
  At this point, we have established an equal-increment partition based DoF distortion model for any object plane at $s_{n}$ when the lens is focused at distance s.

## 4. Calibration Method for Camera Parameters

In close-range photography, the DoF images are seriously distorted, so the calibration accuracy of the distortion parameters is the decisive factor affecting the vision measurement accuracy. When the coupled-calibration method is used to solve the distortion parameters, the estimated errors of intrinsic and extrinsic parameters will be propagated to distortion parameters. Thus, a two-step method is proposed to calibrate the camera parameters, in which distortion parameters are estimated independently.

### 4.1. Independent Distortion Calibration Method Based on Linear Conformation

**Figure 4:**
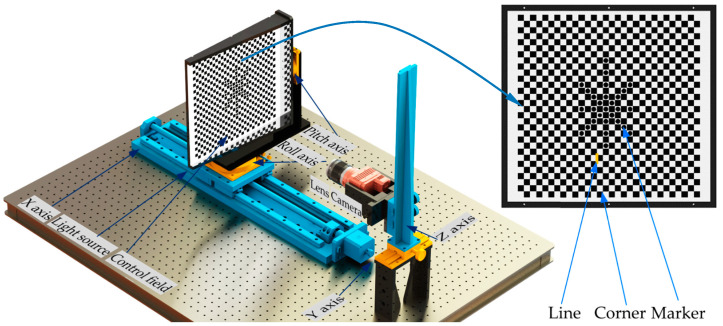
Experimental system for calibrating depth of field (DoF) lens distortion.

Figure 4 details the experimental system for DoF lens distortion, which consists of a monocular camera, a control field, a light source, an electric control platform, and a multi-axis motion controller. The X, Y, A, and C axes of the platform are in the object space, while the Z-axis is in the image space. A control field, with the features of circle, corner, and line, is used to calibrate the lens distortion, and the geometric relationship between the features is known accurately. On this basis, the pose of the control field relative to the image plane can be adjusted by the Perspective-n-Point (PnP) algorithm.

In this paper, the distortion coefficients can be estimated by the plumb-line method [12] alone. It is defined by Brown (1971) as “a straight line in the object space will be mapped to the image plane in a straight way after a perfect lens, and any change of straightness can be reflected as the lens distortion described by the radial and decentering distortion coefficients.”

**Figure 5:**
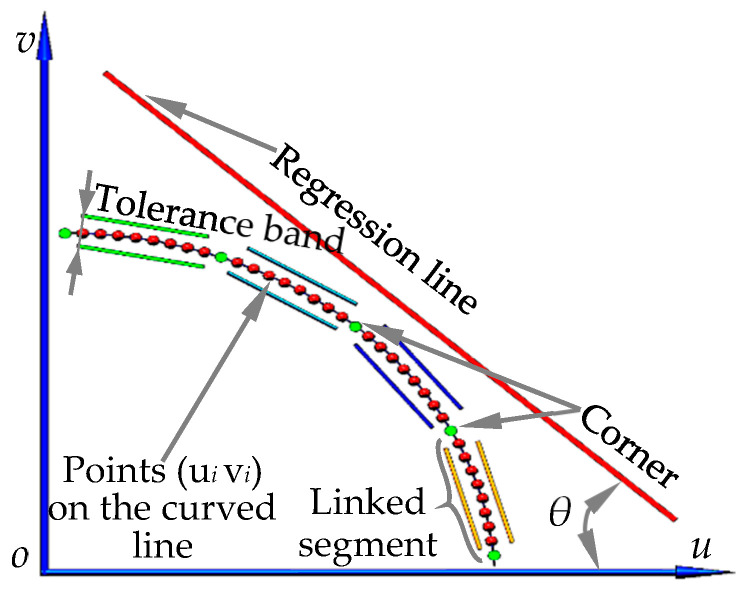
Schematic diagram of distortion calibration based on linear conformation configuration.

As demonstrated in Figure 5, when N edge points $\left(\begin{array}{cc} u_{1} & v_{1} \end{array})\cdots (\begin{array}{cc} u_{N} & v_{N} \end{array}\right)$ on the same curve are known, the regression line equation determined by the point group is

(22) $$\alpha^{\prime }u+\beta^{\prime }v-\gamma^{\prime }=0$$

Let $\alpha^{\prime }=\sin\theta$, $\beta^{\prime }=\cos\theta$, $\gamma^{\prime }=A_{u}\sin\theta +A_{v}\cos\theta$, $\tan2\theta =-\frac{2V_{uv}}{V_{uu}-V_{vv}}$. where θ is the angle between the regression line and the u axis (Figure 5). $A_{u}=\frac{1}{N}\sum_{i=1}^{N}u_{i}$, $A_{v}=\frac{1}{N}\sum_{i=1}^{N}v_{i}$, $V_{uu}=\frac{1}{N}\sum_{i=1}^{N}{\left(u_{i}-A_{u}\right)}^{2}$, $V_{uv}=\frac{1}{N}\sum_{i=1}^{N}\left(u_{i}-A_{u})(v_{i}-A_{v}\right)$, $V_{vv}=\frac{1}{N}\sum_{i=1}^{N}{\left(v_{i}-A_{v}\right)}^{2}$.

Given there are L lines and there are $N_{l}$ points in the l-th line, the average sum of squared distances from the points $\left(\begin{array}{cc} u_{li} & v_{li} \end{array}\right)$ to all the lines can be written as

(23) $$D=\frac{1}{L}\cdot \sum_{l=1}^{L}\frac{1}{N_{l}}\cdot \sum_{i=1}^{N_{l}}{\left(\alpha_{l}^{\prime }u_{li}+\beta_{l}^{\prime }v_{li}-\gamma_{l}^{\prime }\right)}^{2}$$

Any distortion of a line’s straightness in the image plane can be corrected by a mapping involving radial and decentering distortion. Thus, substitute Equation (2) into Equation (23) and we can get

(24) $$F({\bar{u}}_{li},{\bar{v}}_{li};K_{1},K_{2},P_{1},P_{2})=0$$

If there are L lines in an image and $N_{l}$ observation points are extracted from each line, we can have $L\cdot N_{l}$ equations. In these equations, there are L+4 variables (L line coefficients and 4 distortion coefficients). If $L\cdot N_{l}>L+4$, the optimal solution of distortion coefficients can be obtained.

After solving the image distortion coefficients, the inverse mapping $imR(u,v)=imD(u_{d},v_{d})$ between the undistorted image imR and distorted image imD is established by cubic B-spline interpolation. In this way, the image distortion can be corrected. Besides, in this paper, the three straightness indicators of the maximum, average, and root mean square (RMS) $d=\sqrt{D/\sum_{l=1}^{L}N_{l}}$ of the point-to-line distance, and the Peak Signal-to-Noise Ratio (PSNR) $PSNR=10\times \log_{10}({\left(2^{n}-1\right)}^{2}/MSE$, are used to evaluate the distortion correction effects. D has been defined in Equation (23), and MSE is the mean square error of the image before and after distortion correction.

### 4.2. Image Processing and Camera Calibration

**Figure 6:**
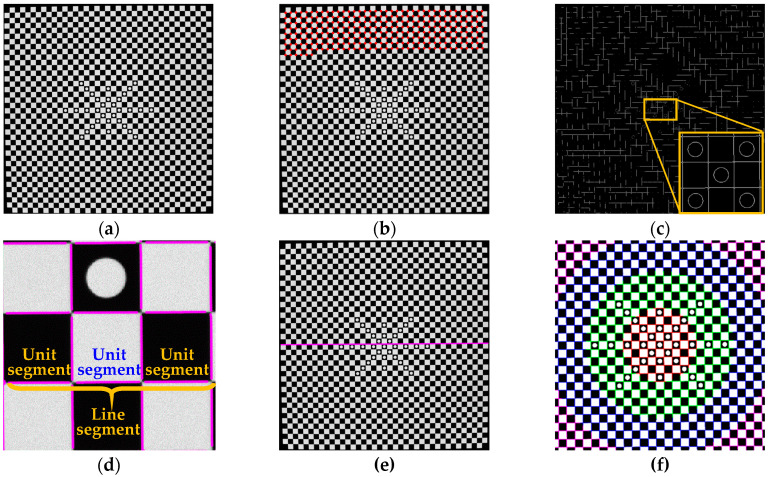
Image processing procedures for linear conformation: (**a**) image of the control field; (**b**) corner detection; (**c**) edge point connection; (**d**) point reselection; (**e**) horizontal line extraction; (**f**) line detection results in different areas.

In this paper, the parameters in the equal-increment partition based DoF distortion model are calculated by using straight lines in a particular area of the control field. To this end, the corner control based method, for extracting line segments within a partition, is proposed. As shown in Figure 6, the image processing procedures include the following:

(1) Image acquisition. Capture the image of the control field using the monocular camera (Figure 6a).
(2) Point detection. Corners of the checkerboard are extracted by the Harris detector (Figure 6b), and the edge points on the curve are detected by the Canny operator with subpixel accuracy.
(3) Point connection. Use the edge points between two adjacent corners to form unit segments (Figure 5). In each segment, David Lowe’s method [37] is used to track and connect the edge points in the four-link area from one particular point to the others (Figure 6c). The minimum connection length is set to be greater than 10 pixels.
(4) Point reselection. The distortion is not evenly distributed on the image, with the largest at the image edge, which makes it difficult to remove the noisy point. To solve this problem, the tolerance band of 4 pixels (Figure 5) set in each unit segment is used as the constraint to filter out the outliers. Consequently, the new edge points are determined (Figure 6d).
(5) Line extraction. Any line can be obtained according to the corner position and the predefined distortion radius. Figure 6e,f shows the extraction results of the 19th horizontal line and the lines in different areas of the control field, respectively.

By combining the image processing results with the DoF distortion partition model, distortion parameters at any position of the DoF can be determined. To avoid the coupling effect between the distortion parameters and other parameters in the camera model, the camera’s intrinsic and extrinsic parameters are preliminarily calibrated by Zhang’s method. Then, we fix the distortion parameters and place the high-precision target in multiple spatial positions to optimize the intrinsic and extrinsic parameters. The cost function to be optimized is

(25) $$\{\begin{array}{c} E_{depth\_dependent}^{q}(R_{q})=\sum_{g=1}^{m_{g}}\left(H^{-1}(u_{0},v_{0},f_{x},f_{y}{,}^{g}K_{i}{,}^{g}P_{j},R_{q},T_{q})\right)\\ i=1,2\\ j=1,2 \end{array}$$

where $E_{depth\_dependent}^{q}(R_{q})$ describes the cost function when the control field is in the q-th pose. $R_{q}$ and $T_{q}$ are the rotation and translation matrices in the q-th pose. Kjg and Pjg are the i-th order radial and j-th order decentering distortion coefficients in the g-th partition of the q-th pose. By using the Levenberg–Marquardt (LM) algorithm, the optimal solution of the camera’s intrinsic and extrinsic parameters can be obtained.

Through the above process, the monocular camera calibration can be realized. In practice, the partition where a spatial point is located ⌈X2+Y2⋅fρ⋅Z⌉ can be determined after estimating its 3D position $\left(\begin{array}{ccc} X & Y & Z \end{array}\right)$. Then, the observed distortion can be corrected by choosing the proper distortion coefficients, thus realizing high-accuracy vision measurements.

## 5. Accuracy Verification Experiments of Both the Distortion Modeling and Calibration Method

### 5.1. Experimental Verification of the 2D Distortion Partitioning Method

**Figure 7:**
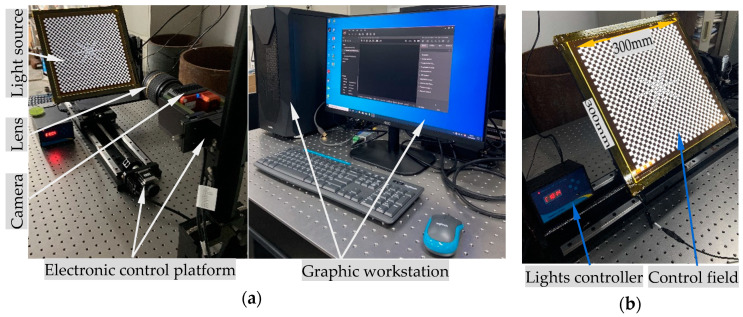
Experimental system for DoF distortion calibration: (**a**) system hardware; (**b**) control field.

The experimental system is shown in Figure 7. The stroke of the electric control platform along the optical axis of the camera is 500 mm, and the size of the control field is 300 × 300 mm. The SIGMA zoom lens (18–35 mm) and HIK ROBOT camera (MV-CH120-10TM) are selected for imaging. The resolution and focal length are set as 2560 × 2560 pixels and 18 mm respectively. The procedures are as follows:

(1) calibrate the intrinsic and extrinsic parameters of the monocular camera;
(2) make adjustments to ensure that the circle features are distributed symmetrically around the image center;
(3) the pose of the control field is determined and adjusted repeatedly to ensure the object and the image planes are parallel;
(4) the control field is driven by the electronic control platform to move several object planes along the optical axis. The image of the control field in each plane is collected and analyzed by the algorithm on the graphic workstation.

**Figure 8:**
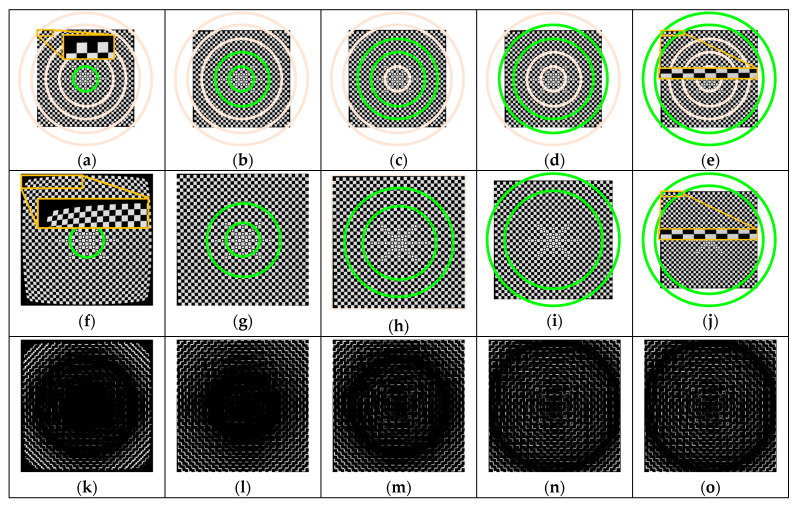
Distortion calibration and correction results based on the equal-radius partition method (f = 18 mm): (**a**) partition 1; (**b**) partition 2; (**c**) partition 3; (**d**) partition 4; (**e**) partition 5; (**f**) distortion correction (partition 1); (**g**) distortion correction (partition 2); (**h**) distortion correction (partition 3); (**i**) distortion correction (partition 4); (**j**) distortion correction (partition 5); (**k**) difference (partition 1); (**l**) difference (partition 2); (**m**) difference (partition 3); (**n**) difference (partition 4); (**o**) difference (partition 5).

**Figure 9:**
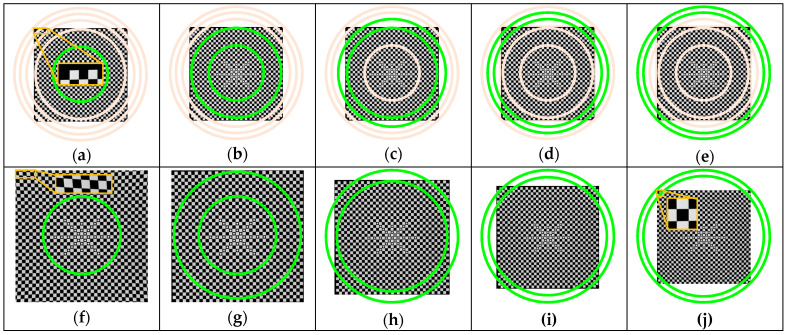

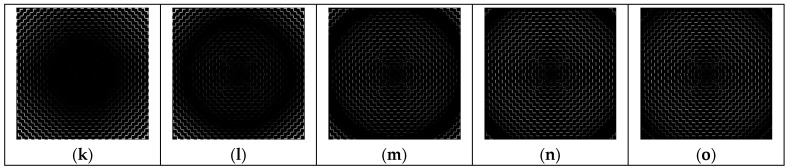
Distortion calibration and correction results based on the proposed partition method (f = 18 mm): (**a**) partition 1; (**b**) partition 2; (**c**) partition 3; (**d**) partition 4; (**e**) partition 5; (**f**) distortion correction (partition 1); (**g**) distortion correction (partition 2); (**h**) distortion correction (partition 3); (**i**) distortion correction (partition 4); (**j**) distortion correction (partition 5); (**k**) difference (partition 1); (**l**) difference (partition 2); (**m**) difference (partition 3); (**n**) difference (partition 4); (**o**) difference (partition 5).

First, the accuracy of the 2D distortion partitioning method is verified. The image of the control field at the focus distance is divided into five concentric rings by the equal-radius (Figure 8a–e) and equal-increment (Figure 9a–e) distortion partition models, respectively. In each partition, the corresponding lines (green ones) are selected to solve the distortion coefficients and correct the image distortion. For each of the two partitioning methods, five corrected images can be obtained (i.e., Figure 8f–j and Figure 9f–j). Here, we use Figure 8f and Figure 9f as an example to illustrate the results of distortion correction. The distortion on the image edge solved by the distortion coefficients of the first partition is far beyond the actual distortion here. After distortion correction, distortion is removed overly, thus resulting in the distortion in the opposite direction.

To compare the distortion correction effect of each partition in the image, we subtract the simulated undistorted image with the corrected image, and we get Figure 8k–o and Figure 9k–o. Obviously, the smaller the gray value is, the closer the undistorted image is to the ground truth, and the better the distortion removal effect is. As can be seen from the figures, the distortion correction results of each partition by the equal-radius partitioning method (Figure 8k–m) were not as good as that by the equal-increment partitioning method. Notably, in the fourth partition and the fifth partition located at the edge of the image, the green concentric ring in Figure 8n–o had a larger gray value, while in Figure 9n–o the gray values of pixels in the green concentric ring were approximately 0. This shows that the equal-increment partitioning method had a better performance on eliminating distortion.

**Figure 10:**
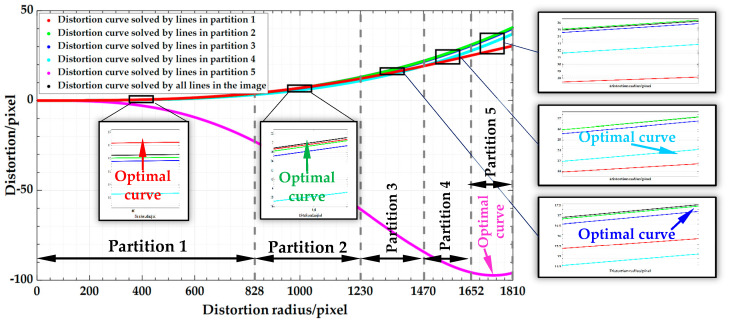
Distortion curves solved by the lines in each partition using the proposed partition method.

Meanwhile, all the lines were used to solve and correct the image distortion as well. Then, the distortion correction effects with and without partition were compared using the aforementioned indexes (Section 4.1). As shown in Table 1, the undistorted images obtained by the two distortion partition methods had a good PSNR of up to 37.61 dB. Compared with the results obtained when without partition, the two partition methods showed a smaller straightness error in each partition. However, compared with the two partitioning methods, the maximum and average errors in the fourth and fifth partitions by the equal-radius partitioning method were at least 4 times and 2 times those by the partitioning method proposed in this paper. That is to say, with the equal-increment partitioning method, each partition can get better distortion correction results. The enlarged image of the best distortion curve for each partition is shown in Figure 10, which validates the effectiveness and accuracy of the proposed partitioning method in 2D settings.

### 5.2. Accuracy Verification Experiments of DoF Distortion Partitioning Model and Camera Calibration

In this section, the accuracy of the DoF distortion model and camera calibration is verified. The control field is driven to move four different object planes within the DoF, two of which are at the limit positions of the front and rear DoF, and the other two planes are within the DoF. The front object plane was divided into five areas with equal distortion increment of 20.2 pixels. Then, based on the distortion parameters in two object planes with known depths, the distortions in the other two object planes are calculated by the non-partition model, the proposed DoF distortion model with equal-radius partition, and the proposed DoF distortion model with equal-increment distortion partition, respectively. Thereafter, we manually adjusted the ring to focus the lens on the two object planes located at the limit positions of the front and rear DoF. Then, based on the calculated radial and decentering distortion coefficients on the two focal planes, Brown’s model [12] with equal-radius partition is used to estimate distortion parameters on the two planes within the DoF.

Furthermore, the results are compared with the distortion directly solved by the lines (the observed value) within the corresponding partition. To compare the accuracy of different DoF distortion models, we took the in-plane point located in the common area (the second column of Table 2) partitioned by the two models at the same object distance (e.g., 400 mm in the first column of Table 2) as an example. As shown in Table 2, for Brown’s model [12] with equal-radius partition, the maximum and average absolute differences between the calculated and the observed values were 7.32 μm and 2.81 μm, respectively. Those errors are smaller than that of the traditional Zhang’s model without considering the DoF and distortion partition, but much larger than those of the proposed DoF distortion model with equal-radius partition and the proposed DoF distortion model with equal-increment distortion partition, respectively. The maximum and average absolute differences between the calculated and the observed values of the equal-increment distortion partition based DOF distortion model were 1.53 μm and 0.88 μm, respectively. By contrast, the errors of equal-radius partition based DOF distortion model were 4.64 μm and 1.94 μm, which was more than two times those of the proposed model in this paper. The results verified the accuracy of the DoF distortion partitioning model in 3D settings.

**Figure 11:**
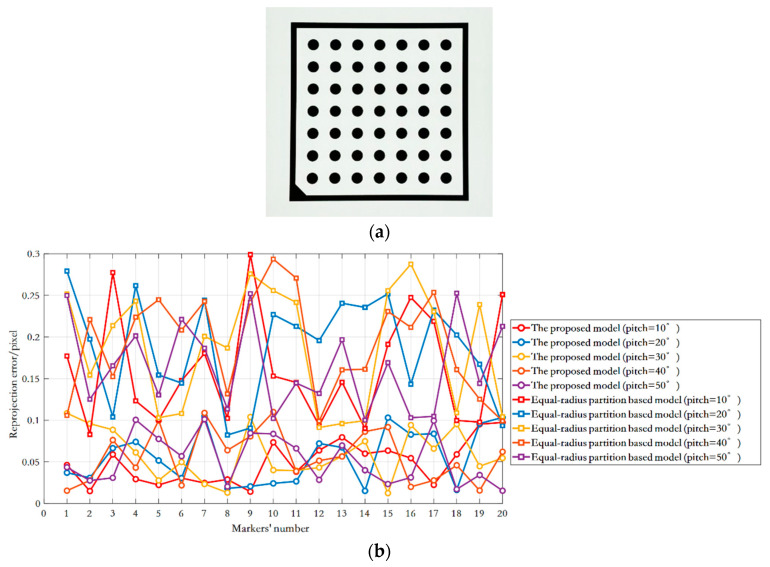

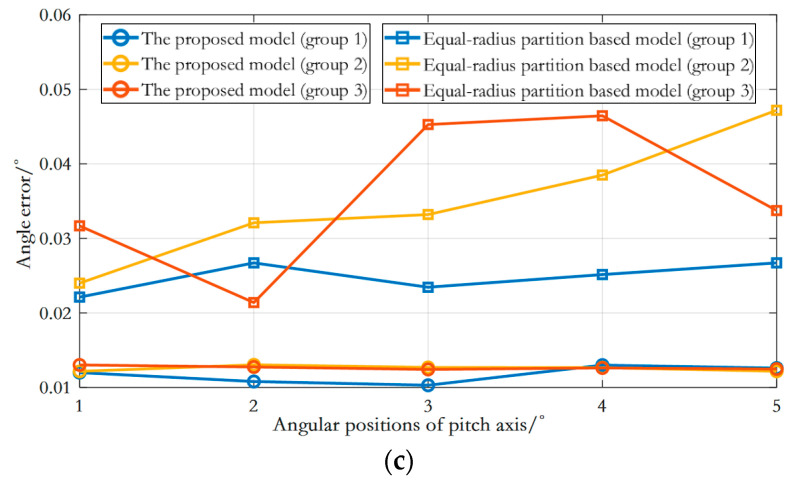
Camera calibration accuracy verification: (**a**) artifact; (**b**) re-projection error; (**c**) angular reconstruction error.

Images of circular markers with known precise distance on the planar artifact are collected. The calibration accuracy of the monocular camera is verified by the re-projection errors and the angular reconstruction errors, respectively. Specifically, the planar artifact is driven by the high-accuracy pitch axis to rotate five positions, between two adjacent ones of which are 10°. In each position, the pose matrix between the planar artifact Figure 11a and the calibrated camera is calculated by the OPNP algorithm [38] with the equal-radius and the equal-increment partitioning based DoF distortion models, respectively. Thereafter, 20 markers are projected back to the image via the estimated pose matrix, and the re-projection errors, the image distances between the projected and observed points, are calculated. As shown in Figure 11b, for equal-radius DoF distortion partition model, the maximum and average re-projection errors of the five positions were 0.29 pixels and 0.17 pixels, respectively, while the maximum and average projection errors of the proposed model were 0.11 pixels and 0.05 pixels, respectively. The angle between two adjacent positions of the artifact is reconstructed with the two models as well. As illustrated in Figure 11c, the 3D measurement accuracy of the system is assessed by comparing it with the nominal angle. The results show that the maximum and average angular errors of equal-radius based DoF distortion partition model were 0.48° and 0.30°, respectively, while those of the proposed model were 0.013° and 0.011°, which means that the angular reconstruction errors are effectively reduced. The above results comprehensively verify the accuracy of the DoF distortion partitioning model and the camera calibration method proposed in this paper.

## 6. Conclusions

This paper has investigated the methods of modeling and calibration of lens distortions for close-range photogrammetry (e.g., short object distance and small focal length). Our work finds that the following:

(1) A focusing-state-independent DoF distortion model is constructed, and the distortion parameters at any object plane can be solved through the distortion on two defocus planes, which removes the human errors introduced by manual adjustment of the focus and zoom rings.
(2) A 2D-to-3D equal-increment partitioning method for lens distortion is proposed. After fusing with the DoF distortion model to form a DoF distortion partition model, the accuracy of lens distortion characterization is further improved.
(3) A two-step method is proposed to calibrate camera parameters, in which the DoF distortion is calculated independently by the plumb-line method, which eliminated the coupling effect among the parameters in the camera model.
(4) Experiments were performed to verify the accuracy of the 2D distortion partition model, DoF-dependent distortion partition model, and camera calibration. The results show that the maximum and average angular reconstruction errors by the proposed model were 0.013° and 0.011° respectively, which validates the accuracy and feasibility of the equal-increment partitioning based DoF distortion method.

The main limitation of the present study is that the number of partitions is not optimized to achieve higher calibration accuracy. Our future work will focus on this and extend our model to other optical systems with fisheye or catadioptric lenses.

## Figures and Tables

**Table 1:** Comparison of distortion correction of the two partition models.

Indicator	Equal-Radius Partition Model/The Proposed Model					Non-Partitioned Model
	Partition 1	Partition 2	Partition 3	Partition 4	Partition 5	
Maximum error/pixel	0.32/0.22	0.62/0.41	0.77/0.53	2.1/0.56	2.7/0.55	7.46
Average error/pixel	0.05/0.03	0.07/0.06	0.10/0.08	0.17/0.08	0.26/0.10	0.52
RMS/pixel	0.04/0.04	0.08/0.06	0.10/0.07	0.11/0.08	0.32/0.09	0.48
PSNR/dB	37.61/37.61	37.26/37.26	37.10/37.30	37.28/37.29	37.34/37.33	37.53

**Table 2:** Accuracy verification for DoF distortion partition model.

Position of Object Plane (mm)	In-Plane Position (Distance from the Distorted Point to the Optical Axis)	Distortion								
		Observed Value (μm)	Brown’s Distortion Model with Equal-Radius Partition		Equal-Radius Partition Based DOF Distortion Model		Equal-Increment Distortion Partition Based DOF Distortion Model		Zhang’s Model	
			Calculated (μm)	Difference |C−O| (μm)	Calculated (μm)	Difference |C−O| (μm)	Calculated (μm)	Difference |C−O| (μm)	Calculated (μm)	Difference |C−O| (μm)
400	Point in partition #1 (56 mm)	86.41	86.33	0.08	86.38	0.03	86.4	0.01	86.27	0.14
	Point in partition #2 (112 mm)	1836.23	1834.12	2.11	1835.82	0.41	1836.09	0.14	1832.42	3.81
	Point in partition #3 (168 mm)	3586.06	3582.81	3.25	3583.7	2.36	3584.84	1.22	3581.81	4.25
	Point in partition #4 (224 mm)	5335.88	5332.01	3.87	5333.06	2.82	5334.57	1.31	5329.22	6.66
	Point in partition #5 (280 mm)	−7085.71	−7093.03	7.32	−7090.35	4.64	−7087.05	1.34	−7095.03	9.32
500	Point in partition #1 (70 mm)	51.3	51.28	0.02	51.28	0.02	51.29	0.01	51.28	0.02
	Point in partition #2 (140 mm)	1468.98	1467.93	1.05	1468.5	0.48	1468.44	0.54	1466.8	2.18
	Point in partition #3 (210 mm)	2886.67	2884.63	2.04	2884.27	2.40	2885.46	1.21	2883.11	3.56
	Point in partition #4 (280 mm)	4304.35	4301.18	3.17	4301.64	2.71	4302.82	1.53	4299.8	4.55
	Point in partition #5 (350 mm)	−5722.03	−5727.29	5.26	−5725.59	4.56	−5723.55	1.52	−5729.84	7.81

## Abbreviations

(List of abbreviations and symbols present in this article)

**Acronym or Symbols**	**Definition**	**Acronym or Symbols**	**Definition**
DoF	depth of field	$K_{i}^{s,s_{n}}$	i-th radial distortion coefficient of the object plane at distance $s_{n}$ when the lens is focused at the distance of s
FoV	field-of-view	$K_{i}^{s,s_{m}}$	i-th radial distortion coefficient of the object plane at distance $s_{m}$ when the lens is focused at the distance of s
$p\left(\begin{array}{cc} u_{li} & v_{li} \end{array}\right)$	undistorted coordinates	$K_{i}^{s,s_{k}}$	i-th radial distortion coefficient of the object plane at distance $s_{k}$ when the lens is focused at the distance of s
$O_{w}X_{w}Y_{w}Z_{w}$	world coordinate system	$\delta P^{s,s_{m}}$	decentering distortion function in the defocused plane at the object distances of $s_{m}$ when the lens is focused at distance s
ouv	image coordinate system	$\delta P^{s,s_{k}}$	decentering distortion function in the defocused plane at the object distances of $s_{k}$ when the lens is focused at distance s
$O_{C}$	optical center	$\delta P^{s,s_{n}}$	decentering distortion function in the defocused plane at the object distances of $s_{n}$ when the lens is focused at distance s
$z^{\prime }$	scaling factor	$P^{s,s_{n}}$	i-th decentering distortion coefficient of the object plane at distance $s_{n}$ when the lens is focused at the distance of s
K	intrinsic parameter matrix	$P_{i}^{s,s_{m}}$	i-th decentering distortion coefficient of the object plane at distance $s_{m}$ when the lens is focused at the distance of s
CCD	Charge Coupled Device	$P_{i}^{s,s_{k}}$	i-th decentering distortion coefficient of the object plane at distance $s_{k}$ when the lens is focused at the distance of s
CMOS	Complementary Metal-Oxide Semiconductor	$\delta_{max}$	maximum value of image distortion
M	transformation matrix	$r_{max}$	maximum distortion radius of the image
VCS	vision coordinate system	$I_{l}$	length of the image
R	rotation matrix	$I_{h}$	height of the image
T	translation matrix	$\delta_{limited}$	minimum distortion value
$\left(\begin{array}{cc} {\bar{u}}_{li} & {\bar{v}}_{li} \end{array}\right)$	distorted coordinates	$r_{limited}$	minimum value of the image distortion radius
$\delta_{u_{li}}$	distortion function of an image point in the u direction	$n_{p}$	number of partitions
$\delta_{v_{li}}$	the distortion function of an image point in the v direction	$\delta_{equ}$	distortion increment
$\left(u_{0},v_{0}\right)$	distortion center	ρ	partition radius
r	distortion radius	$P_{m}$	point in VCS
$K_{1}$	first-order coefficient of radial distortion	$P_{k}$	point in VCS
$K_{2}$	second-order coefficient of radial distortion	$\Pi_{m}$	m-th object plane in the VCS
$P_{1}$	first-order coefficient of decentering distortion	$\Pi_{k}$	k-th object plane in the VCS
$P_{2}$	second-order coefficient of decentering distortion	$\Pi_{n}$	n-th object plane in the VCS
$\delta r_{\infty }$	radial distortion for a lens that is focused on plus infinity	Kis,smg	i-th order radial distortion coefficient in the g-th partition of object plane $\Pi_{m}$
$\delta r_{-\infty }$	radial distortion for a lens that is focused on minus infinity	Pis,smg	i-th order decentering distortion coefficient in the g-th partition of object plane $\Pi_{m}$
$m_{s}$	vertical magnification in the focal plane at object distance s	Kis,skg	i-th order radial distortion coefficient in the g-th partition of object plane $\Pi_{k}$
$\delta r_{s_{}}$	lens radial distortion in the focal plane	Pis,skg	i-th order decentering distortion coefficient in the g-th partition of object plane $\Pi_{k}$
$\delta r_{s_{m}}$	radial distortion in the focal plane when the lens is focused on the distance of $s_{m}$	Kis,sng	i-th order radial distortion coefficient in the g-th partition of object plane $\Pi_{n}$
$\delta r_{s_{k}}$	radial distortion in the focal plane when the lens is focused on the distance of $s_{k}$	Pis,sng	i-th order decentering distortion coefficient in the g-th partition of object plane $\Pi_{n}$
f	focal length	PnP	Perspective-n-Point
$K_{i}^{s}$	i-th radial distortion coefficient for focused object plane at distance s	θ	angle between the regression line and the u axis
$K_{i}^{s_{m}}$	i-th radial distortion coefficient when the lens is focused on the distance of $s_{m}$	D	average sum of squared distances from the points $\left(\begin{array}{cc} u_{li} & v_{li} \end{array}\right)$ to all the lines
$K_{i}^{s_{k}}$	i-th radial distortion coefficient when the lens is focused on the distance of $s_{k}$	imR	undistorted image
$\delta r_{u}$	component of the decentering distortion in u direction	imD	distorted image
$\delta r_{v}$	component of the decentering distortion in v direction	RMS	root mean square
$K^{s,s_{p}}$	radial distortion coefficient in the defocused plane with the depth of $s_{p}$ when the lens is focused at distance s	PSNR	Peak Signal-to-Noise Ratio
g	empirical coefficient	MSE	mean square error of the image before and after distortion correction
$K^{s_{p}}$	radial distortion coefficient in the focal plane at distance $s_{p}$	$R_{q}$	rotation matrix in the q-th pose
$K^{s}$	radial distortion coefficient in the focal plane at distanced s	$T_{q}$	translation matrix in the q-th pose
$\delta r^{s,s_{n}}$	radial distortion function of the object plane at distance $s_{n}$ when the lens is focused at the distance of s	Kig	i-th order radial distortion coefficient in the g-th partition of the q-th pose
$\delta r^{s,s_{m}}$	radial distortion function of the object plane at distance $s_{m}$ when the lens is focused at the distance of s	Pjg	j-th order decentering distortion coefficient in the g-th partition of the q-th pose
$\delta r^{s,s_{k}}$	radial distortion function of the object plane at distance $s_{k}$ when the lens is focused at the distance of s	LM	Levenberg-Marquardt
